# COVID-19 and adaptive behavior of returns: evidence from commodity markets

**DOI:** 10.1057/s41599-022-01332-z

**Published:** 2022-09-21

**Authors:** Muhammad Naeem Shahid

**Affiliations:** Lyallpur Business School, Government College University Faisalabad, Chiniot Campus, Pakistan

**Keywords:** Finance, Business and management

## Abstract

This study examines the Adaptive Market Hypothesis during the COVID-19 pandemic. The pandemic has impacted global economic activity, trade, and financial market activity. There has been much interest in testing financial market theories and relationships during COVID-19. Therefore, we have investigated the varying return predictability from commodities during COVID-19 concerning the adaptive market hypothesis. By applying linear and non-linear econometric models, we find a strong engagement of adaptive behavior of returns from commodities during the ongoing pandemic. The inconsistent return behavior is facilitated by dividing the sample period into different phases. Our results indicate that AMH best explains the impact of COVID-19 on commodity markets.

## Introduction

No prior infectious disease has caused such swings in financial market returns as the Covid-19 pandemic.

The outbreak has caused havoc around the globe, with millions of people affected; precautionary measures instituted, resulting in reduced economic activity. Halted. The massive spread of the virus compelled WHO to declare COVID-19 firstly as a global emergency on February 20, 2020, and later on a pandemic on March 11, 2020. The current volatility levels of financial markets mirror the expected damages as pr studies (Al-Awadhi et al., 2020; Ashraf, 2020; Liu et al., 2020; Onali, 2020). Major stock markets observed a double-figure decline and a 30% decline posted by S&P 500 in just 16 trading days. Baker et al. (2020) reported that the spread of the Coronavirus from China to other countries and the resulting pandemic had caused a fall in returns and higher volatility in the financial markets.

In the United States, current volatility levels rival those last seen in 2008, 1987, and the early 1930s (Baker et al., 2020). The changes in the level of predictability of the financial markets cast serious doubt about the validity of the Efficient Market Hypothesis (EMH), proposed by Fama (1970)^1^. EMH states that investors cannot forecast the changes in the prices based on past trading information of the securities. While some studies support EMH, many found that security prices are predictable as market conditions induce levels of predictability in security returns. Also, the AMH of Lo (2004) explains well the changes in the level of return predictability, combining the concepts of EMH and behavioral finance.

Lo (2004) explains that financial markets have episodes of varying performance, efficiency, and volatility over time. Also, investors’ preferences change over time, forced by adoption, where past price movements dictate current prices, thus contradicting EMH. Therefore, the predictability of returns exhibits cyclic patterns due to information technology, macroeconomic institutions, and market regulations and policies, thus, indicating the mark of adaptive market hypothesis (AMH). Moreover, Lo (2004) claims that markets evolve due to panics, bubbles, and crises. Recently financial markets have been through Covid-19-related developments. These developments are the primary motivation to conduct the current study. A year before, no one had thought of the lifestyle we currently have. Researchers’ attention has shifted towards Covid-19-induced volatility and financial market performance.

We have selected this topic since the Covid-19-induced shocks in financial markets could validate AMH. This hypothesis states that markets go under phases of varying predictability depending upon market conditions. Similarly, COVID-19 encompasses a few episodes (Wagner, 2020) during the first phase of the pandemic, which is the incubation phase where only sophisticated and well-informed investors partially anticipate the future of financial markets and trends of investments. The second phase (the outbreak phase) exhibits growth in investors’ attention towards Coronavirus discussion through Google search and conference calls. Finally, investors realize that the virus could affect them directly and that financial markets swing widely in the fever phase. We have used data of popular commodities traded on the Chicago Board of Options Exchange (CBOE), USA starting with COVID-19.

We run our analysis in two parts. First, we apply the Generalized Spectral (GS) test to detect whether commodity markets exhibit adaptive behavior during COVID-19. If so, then secondly, we apply linear and non-linear econometric models to identify whether evolving behavior of markets produces a prediction for profitable opportunities during the pandemic. Therefore, we have divided the sample period into Epidemic, Pandemic & Endemic periods, and fatality periods (explained in the “Methods” section). We find that the returns from commodities traded on (CBOE) go under predictability periods and no predictability. Thus, shocks in the behavior of returns are engaged and best explained by AMH because predictability from commodities possesses linear and non-linear dependence that arises and disappears during COVID-19 sub-sample phases.

This study enhances the existing literature on the engagement of COVID-19 shocks with the adaptive market hypothesis in several ways. Firstly, this study investigates the engagement of evolving behavior of commodity indices with AMH during the COVID-19 crisis as the Adaptive Market Hypothesis permits the returns from investment in commodities to fluctuate over time. Secondly, most of the research during COVID-19 facilitates the behavior of equity markets, but no research considers commodity markets which may provide a variety of findings. Thirdly, the current study is first of its nature, which investigates the behavior of returns from commodities under different prevailing COVID-19 conditions to elucidate which of the conditions prove more conducive to the performance of these commodities. This study suggests that returns from financial commodity markets manifest adaptive behavior during COVID-19 and engage with the shocks produced by phases of COVID-19. The structure of the thesis is as follows: we present the literature in the next section, while data description and methodology are in the section “Methods”. Section “Results” deals with data analysis, while we present the conclusion and discussion in section “Conclusion”.

## Literature review

COVID-19 has produced a swing in the economies and financial markets of the world. The epidemic (Li et al., 2020) and pandemic (Ashraf, 2020) phases of COVID-19 initiate a swing in economic activities as the lives of billions of people are affected by the disease spread around the globe (Dunford et al., 2020). According to Gates (2020), severe outbreaks of COVID-19 have badly affected the countries like China, Iran, Italy, France, Spain, the UK, and the USA. Many studies have reported that viral diseases badly affect the economies through financial market crashes during the infectious periods (see, for example, Lee and McKibbin, 2004; Chen et al., 2007; Baker et al., 2012; Bai, 2014; Macciocchi et al., 2016; Del-Giudice and Paltrinieri, 2017; Chen et al., 2018). Goodell (2020) has conducted a pioneer study that presents a survey on comprehensive literature displaying the economic impact of natural calamities like climate changes, wars, and localized disasters. It reveals that the COVID-19 pandemic has caused damage to local and global economies and is associated with the underperformance of the financial markets, banking, and insurance sector. He further explains that the COVID-19 era is plentiful for future research in financial markets.

Similarly, Al-Awadhi et al. (2020) provide evidence of a direct linkage between the impact of COVID-19 and stock market returns. By employing firm-level data from China, they examine the early impact of the COVID-19 outbreak on share prices in China. They find a significant negative relationship between the increasing number of death cases and returns from the share of companies under study. Liu et al. (2020) investigate the short-term impact of the covid-19 outbreak on financial market indices from 21^2^ countries. They find that the UK, Italy, Germany, USA, Singapore, Korea, and Japan are the primary affected countries by COVID-19. Onali (2020) uses GARCH (1,1) model to uncover the impact of deaths resulting from COVID-19 on Dow Jones and S&P500 indices of the US and six other countries^3^. The study finds no impact of deaths on financial market volatility in the US apart from deaths reported in China. Likewise, Zhang et al. (2020) found that COVID-19 has led to an increase in global financial market risk. Ashraf (2020) investigated the period from January 22, 2020, to April 17, 2020, to examine the impact of confirmed death cases in the financial markets of 64 Countries. He finds a negative relationship between the death rate and volatility of financial markets, exhibiting returns decrease as the death cases increase. Ali et al. (2020) investigate the impact of COVID-19 on financial market indices like bitcoin, bond index, equity, and commodity indices. With the application of the E-GARCH model, they investigate the volatility in said indices in different sample periods and find that the markets of china remain stable.

Baker et al. (2020) investigate the pandemic impact of COVID-19 on U.S. financial markets and compare the impact of COVID-19 with past pandemics. They find that restrictions imposed by the government on commercial activities and voluntary social distancing during COVID-19 had a very forceful impact on the volatility of U.S. financial markets compared to past infectious disease pandemics of 1918–19, 1957–58, and 1968. Alfaro et al. (2020) use data from the US and find that equity market value declines in response to pandemics such as Covid-19 and SARS. In contrast, global markets show more volatile behavior during different phases selected in the study. Papadamou et al. (2020) use the google trend synthetic index to uncover the impact of the pandemic on the financial markets of Asia^4^, Australia, Europe^5^, Russia, the UK, and the US. They find that the Term COVID-19 search queries and google based anxiety of COVID-19 directly impact the volatility of financial markets due to pandemic conditions with more intensity in European countries than in the other sample countries. However, an increasing trend is observed in the average volatility of stock markets in the US, the UK, and Germany while COVID-19 moves from epidemic to pandemic. Shahid et al. (2020a) find that commodity markets go under periods of significant predictability and no predictability due to various crises prevailing in the market, which supports AMH. Okorie and Lin (2021) find the fractal contagion effect of the COVID-19 pandemic on the stock markets. Gunay (2021) compares COVID-19 with the Global Financial Crisis (GFC) from currency markets. He finds the shockwave of the pandemic in the total volatility spillover is about eight times greater than that of the GFC.

Lin and Su (2021) capture the behavior of returns from energy commodities and find that commodity markets go under tremendous changes during the pandemic. Zaremba et al. (2021) investigated 49 countries and found the limited impact of government interventions (Workplace and school closures) on stock market liquidity. Okorie and Lin (2021) investigate the impact of the pandemic on four major financial markets from the most affected counties (Brazil, India, Russia, and the USA). They find no variation in the behavior of the returns from Brazile and the USA markets. Due to the pandemic, they find inefficiency in India and efficiency in Russian financial markets. Al-Refai et al. (2022) use the Kalman filter to investigate the shocks of COVID-19 and oil prices on GCC financial markets. They find that except in Oman, all the GCC financial markets react positively and negatively to oil price changes and COVID-pandemics with a higher magnitude after March 2020. Matos et al. (2021) explore the relationship between confirmed death cases during COVID-19 in Hubei, China, and returns from S&P 500 index. Using partial-coherencies for Granger-causality in the quantiles approach, they find a negative impact of death rate on returns from the S&P-500 index.

## Methods

We select the most popular commodities traded on the Chicago Board of Options Exchange (CBOE), which belong to three vital sectors of the economy, Agriculture, Energy, and Precious metals. We have used DataStream for data collection. The data spans from December 2, 2019, to December 31, 2020.

If the markets are adaptive, the behavior of returns from commodities should go under periods of independence and dependency (see Shahid and Mehmood, 2015; Shahid and Sattar, 2017; Shahid et al., 2018, 2020b). To explore the time-varying behavior of the return from commodities, we divide the sample period into different sub-sample, firstly, the COVID-19 Epidemic, Pandemic (based on Haroon and Rizvi, 2020) Endemic periods. Secondly, periods comprising COVID-19 fatalities in China, Europe, and the USA (based on Al-awadhi et al., 2020; Haroon and Rizvi, 2020). Thus, we name the three fatality periods as Fatal1 (fatalities in China), Fatal2 (fatalities in Europe), and Fatal3 (fatalities in the USA and around the globe).

The epidemic phase ranges from the emergence of the Coronavirus to March 10, 2020, when COVID-19 was rapidly spreading in China. The Pandemic Period is around March 10, 2020 (as per WHO) to August 2020, while the period after August 2020 is endemic. Fata1 phase ranges from January 1, 2020, to February 14, 2020, Fatal2 spans from February 15 to February 28, 2020, and Fatat3 entails the post-Fatal2 period (as per WHO). We have calculated the returns from the commodity series using the formula: *r*_*t*_ = [*ln*(*P*_*t*_) − *ln*(*P*_*t*−1_)] × 100 to apply linear and non-linear econometric models. Whereas time *t*, the natural logarithm of returns are represented by *ln*(*P*_*t*_), while at *t*−1, the natural logarithm is *ln*(*P*_*t*−1_). We have presented summary statistics of log returns in Table 1 for the entire sample and its phases. The descriptive statistics for agriculture indices show that during (COVID 19), only Feeder Cattle and Live Cattle produce negative returns (see Table 1). While all other indices generate positive returns, agriculture and soybean generate the highest positive return. Most energy indices produce negative returns, while all precious metals produce positive returns during COVID-19.

**Table 1:** Statistics for Returns from Commodities are presented in columns from 2–5 for full-sample, and Mean & standard deviation (SD) are presented for Epidemic, Pandemic, and Endemic Phases.

	Full sample period COVID-19				Epidemic		Pandemic		Endemic	
	Mean	SD	Max	Min	Mean	SD	Mean	SD	Mean	SD
*Agriculture indices*										
Agr-Liv-Stock	0.07	0.91	2.77	−3.56	−0.07	0.8	−0.06	1.09	0.24	0.76
Agriculture	0.1	0.94	3.39	−2.92	−0.03	0.78	−0.05	1	0.29	0.97
Coffee	0.02	2.38	6.29	−7.37	−0.1	2.85	−0.06	2.41	0.08	2.04
Cotton	0.07	1.44	5.18	−5.47	−0.09	1.31	−0.03	1.8	0.21	1.11
Cocoa	0.01	1.73	5.26	−5.08	0.02	1.47	−0.16	1.93	0.08	1.7
Corn	0.09	1.28	4.24	−3.55	−0.02	1.09	−0.16	1.37	0.41	1.25
Feeder cattle	−0.01	1.59	7.48	−5.87	−0.15	1.23	0.11	2.21	−0.04	1.02
Live cattle	−0.04	1.63	5.46	−5.82	−0.32	1.21	0.04	2.31	0.07	1.02
Soybean	0.16	0.98	3.15	−3.24	0.01	0.79	0.03	0.98	0.37	1.07
Sugar	0.08	1.84	5.62	−5.36	−0.02	1.42	−0.1	2.24	0.2	1.59
Wheat	0.07	1.53	5.13	−3.59	−0.04	1.2	0.04	1.65	0.18	1.61
*Energy indices*										
Red-Energy	0.03	1.52	5.17	−8.6	−0.28	1.54	0.06	1.99	0.18	0.91
Light Energy	0.04	1.11	3.72	−5.64	−0.19	1.12	0.04	1.43	0.18	0.73
Heating oil	−0.09	3.3	10.96	−17.7	−0.63	3.04	0.02	4.44	0.19	2.11
Petroleum	−0.07	4.48	17.37	−33.2	−0.7	3.8	0.14	6.46	0.17	2.23
Unld-Gaso	−0.05	4.68	19.31	−26.3	−0.47	3.39	0.09	6.98	0.18	2.37
Nonn-Gas	−0.01	2.14	7.2	−12.9	−0.39	2.12	0.08	2.87	0.18	1.21
Non-Ener	0.07	0.71	2.31	−3.02	−0.04	0.61	0.02	0.87	0.18	0.6
Crude Oil	−0.06	6.54	32.01	−56.8	−0.73	4.27	0.16	10.19	0.18	2.4
*Precious metals*										
Gold	0.1	1.32	5.61	−5.06	0.19	1	0.16	1.6	−0.05	1.23
Metal	0.11	1.41	5.72	−5.43	0.18	1.03	0.18	1.66	−0.04	1.39
Silver	0.17	2.83	7.25	−12.3	−0.01	1.61	0.32	3.15	0.09	3.09
Copper	0.11	1.36	3.96	−8.1	−0.09	0.95	0.16	1.72	0.18	1.23
Palladium	0.11	3.4	22.94	−23.8	0.37	2.48	−0.02	4.89	0.13	1.98
Platinum	0.07	2.63	11.19	−12.2	−0.05	1.72	0.1	3.52	0.16	2.13
Ind-Metal	0.08	0.98	2.72	−3.39	−0.1	0.82	0.09	1.13	0.16	0.93
Nickel	0.08	1.59	4.29	−4.89	−0.12	1.65	0.08	1.77	0.18	1.42
Zinc	0.08	1.38	3.21	−4.38	−0.21	1.43	0.13	1.44	0.16	1.29

**Fig. 1:**
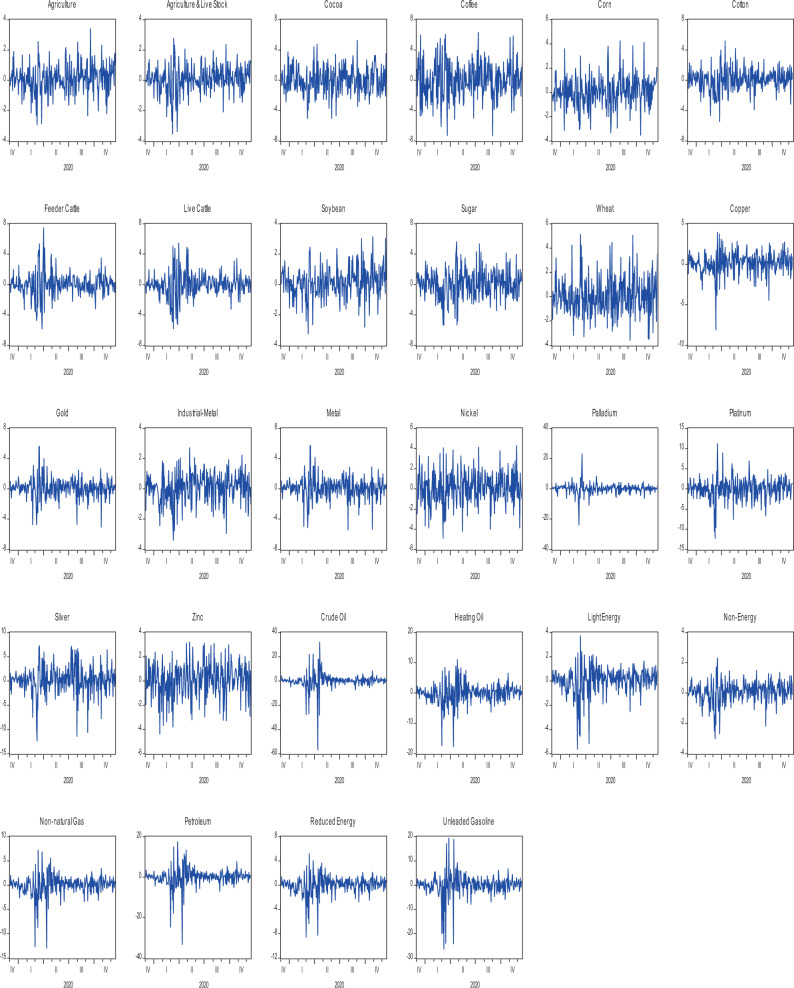
The behavior of returns. It depicts the daily returns captured from different commodities under study.

**Fig. 2:**
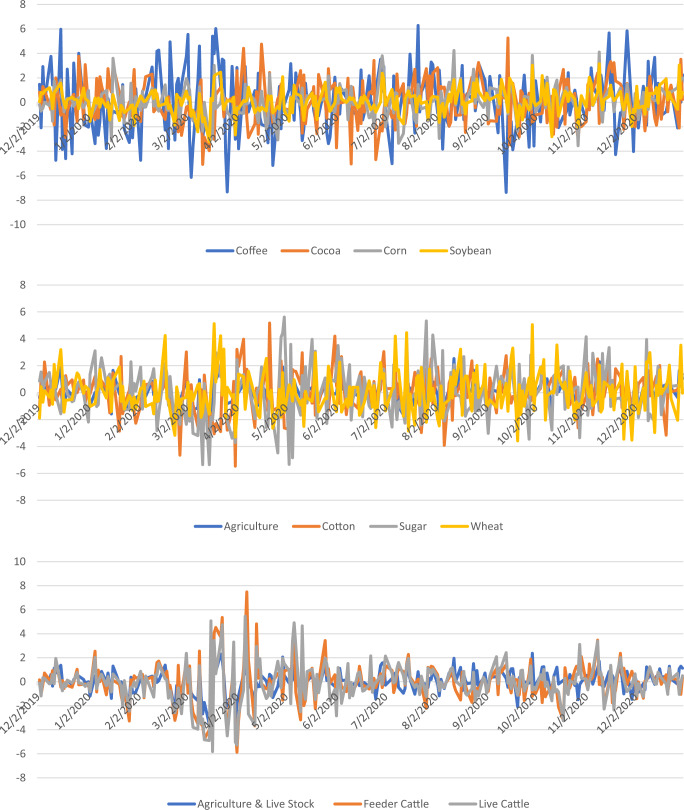
Combined graph for related commodities. The graphs present returns from agriculture sector.

**Fig. 3:**
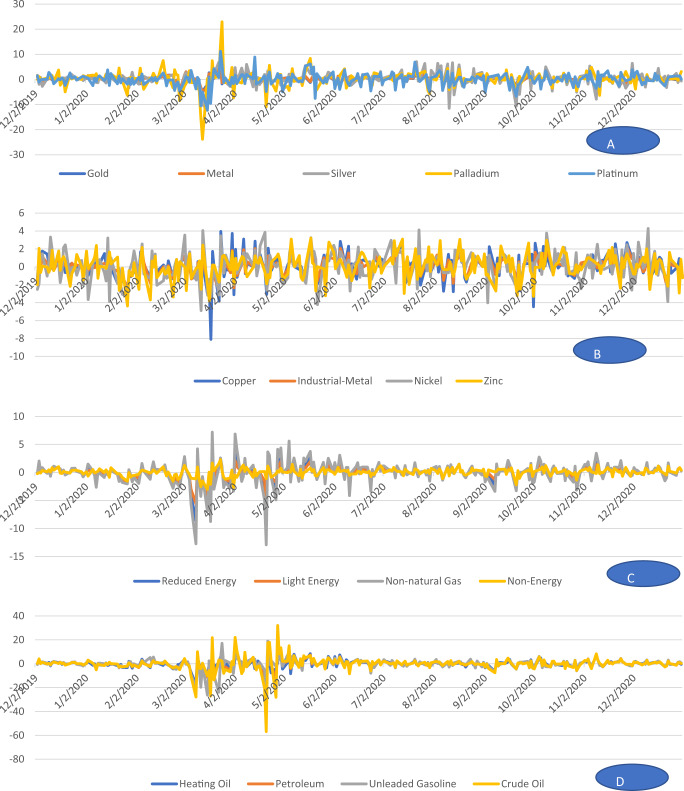
Combined graphs for related commodities. Returns from precious metals are presented in (Graphs A and B) and from energy sector are presented in (Graphs C and D).

On the other hand, most agriculture indices exhibit negative returns during epidemic and pandemic phases, but all the agriculture indices produce positive returns during the endemic except for Feeder cattle. Amazingly during the epidemic phase, all energy indices generate negative returns, this behavior reverts and becomes positive during the pandemic and endemic phases, which is a good sign of AMH. On the other hand, most indices from precious metals produce negative returns in an epidemic while positive returns in the other two phases. Log returns over the entire sample period are presented for each commodity individually in Fig. 1, while Figs. 2 and 3 present the return of related commodities of the three sectors under study.

Based on the study of Ghazani and Ebrahimi (2019), we first apply Generalized Spectral (GS) test with a one-month rolling window to explore the varying behavior of returns from commodities during the COVID-19 crisis. The GS test can capture linear and non-linear dependencies at the same time. Moreover, we apply linear and non-linear tests to identify the degree of predictability (profitable opportunities), and the adaptive behavior of commodities’ return (Urquhart and Hudson, 2013; Shahid et al., 2019a) suggests.

### The Generalized Spectral (GS) test for evolving behavior of indices

To discover the linear and non-linear dependencies, we apply the non-parametric *Generalized Spectral (GS) test* (Escanciano and Velasco, 2006) and relate the time-varying behavior of commodities with levels of predations through linear and non-linear models separately. Let *Y*_*t*_ is stationary time series where the martingale difference sequence cannot forecast the returns in the future, the null hypothesis of a martingale difference sequence is

$${\mathrm{H}}_0:m_0\left( y \right) \,=\, 0\,{{{\mathrm{for}}}}\,{{{\mathrm{all}}}}\,\theta \,\ge\, 1;m_0\left( y \right) = E\left[ {Y_t - \mu } \right]$$

$${\mathrm{H}}_1:P\left( {m_\theta \left( {Y_{t - \theta }} \right)} \right)\, \ne\, \,0\, \,>\, 0\,{{{\mathrm{for}}}}\,{{{\mathrm{some}}}}\,\theta \ge 1.$$

Let $\varphi _0\left( x \right) = E\left[ {\left( {Y_t - \mu } \right){\mathrm{e}}^{ixY_{t - \theta }}} \right]$ be a non-linear gauge of conditional mean dependence where $x \in R$. Consequently, the null hypothesis above is consistent with $\varphi _0\left( x \right) = o$ for all $\theta \ge 1.$ Escanciano and Velasco (2006) apply the generalized spectral distribution function:

$$H\left( {\psi ,x} \right) = \varphi _0\left( x \right)\psi + 2\mathop {\sum}\limits_{\theta = 1}^\infty {\varphi _\theta \left( x \right)} \left[ {\frac{{\sin \left( {\theta \pi \psi } \right)}}{{\theta \pi }}} \right];\psi \in \left[ {0,1} \right].$$

The sample estimate of *H* turns into

$$\hat H\left( {\psi ,x} \right) = \varphi _0\left( x \right)\psi + 2\mathop {\sum}\limits_{\theta - 1}^{n - 1} {\sqrt {\left( {1 - \theta /n} \right)} \hat \varphi _0\frac{{\sin \left( {\theta \pi \psi } \right)}}{{\theta \pi }}}$$

where $\hat \varphi _0 = \left( {n - \theta } \right)^{ - 1}\mathop {\sum}\nolimits_{t = 1 + \theta }^n {\left( {Y_t - \bar Y} \right){\mathrm{e}}^{ixY_{t - \theta }}}$ and $\sqrt {\left( {1 - \frac{\theta }{n}} \right)}$ It is a sample finite correction factor. Therefore, the generalized spectral distribution function under the null of the martingale difference hypothesis (MDH) changes into $\left( {\psi ,x} \right) = \phi _0\left( x \right)\psi$. The test stems from the difference between $\hat H\left( {\psi ,x} \right)$ and $\bar H_0\left( {\psi ,x} \right) = \phi _0\left( x \right)\psi$ as follows:

$$S_n\left( {\psi ,x} \right) = \sqrt {n/2} \left[ {\hat H\left( {\psi ,x} \right) - \hat H_0\left( {\psi ,x} \right)} \right] = \mathop {\sum}\limits_{\theta - 1}^{n - 1} {\sqrt {\left( {n - \theta } \right)} \hat \varphi _0\frac{{\sqrt 2 \sin \left( {\theta \pi \psi } \right)}}{{\theta \pi }}}$$

We use the Cramer–von Mises norm in the equation below to examine the distance of $S_n\left( {\psi ,x} \right)$ of *ψ* and *x*

$$D_n^2 = \mathop {\int}\limits_R {\mathop {\int}\nolimits_0^1 {\left| {S_n\left( {\phi ,x} \right)} \right|^2W({\mathrm{d}}x){\mathrm{d}}\phi } } = \mathop {\sum}\limits_{\theta = 1}^{n - 1} {\left( {n - \theta } \right)\frac{1}{{\left( {\theta \pi } \right)^2}}} \mathop {\int}\limits_R {\left| {\varphi _\theta \left( x \right)} \right|^2W{\mathrm{d}}x}$$

Where the weighting function *W*(.) fulfils some moderate conditions. If the standard normal cumulative distribution function is considered as a weighting function, the following statistics results:

$$D_n^2 = \mathop {\sum}\limits_{\theta = 1}^{n - 1} {\frac{{n - \theta }}{{\theta \pi ^2}}} \mathop {\sum}\limits_{t = \theta + 1}^n {\mathop {\sum}\limits_{s = \theta }^n {\left( {Y_t - \bar Y_{n - \theta }} \right)\left( {Y_s - \bar Y_{n - \theta }} \right)\exp \left[ { - \frac{1}{2}\left( {Y_{t - \theta } - Y_{s - \theta }} \right)^2} \right]} }$$

**Fig. 4:**
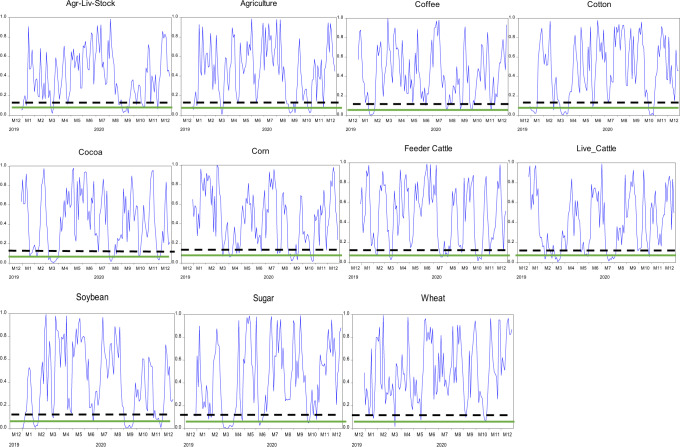
The evolving behavior of *p*-values of the Agriculture Commodities using one-month window length for GS test-statistic. Black dashed-line and green-line present Levels of significance at 10% and 5%, respectively.

**Fig. 5:**
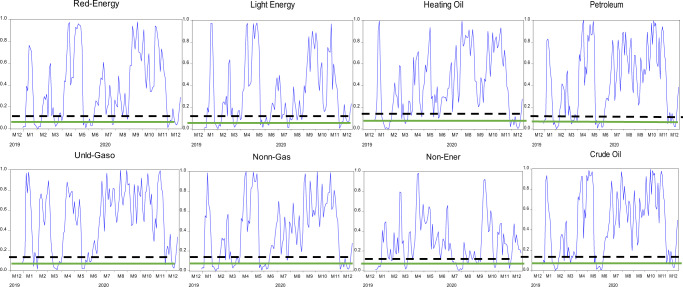
The evolving behavior of *p*-values of the energy commodities using one-month window length for GS test-statistic. Black dashed-line and green-line present Levels of significance at 10% and 5%, respectively.

**Fig. 6:**
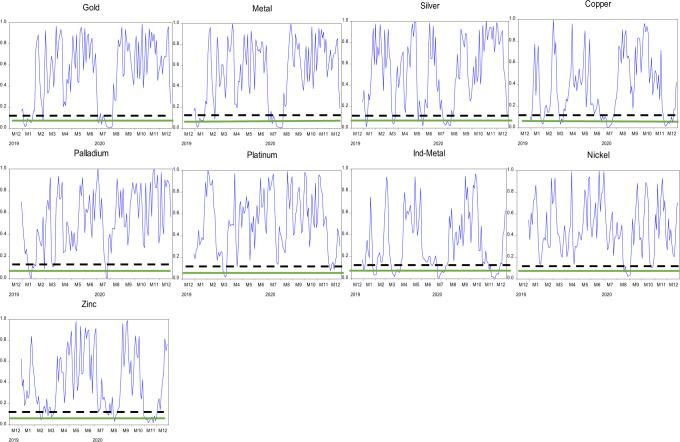
The evolving behavior of *p*-values of the precious metals using one-month window length for GS test-statistic. Black dashed-line and green-line present levels of significance at 10% and 5%, respectively.

The null hypothesis of the martingale difference hypothesis is rejected when values of $D_n^2$ are substantial. The *p*-values of the test statistic $D_n^2$ are acquired by the steps determined in the study (Escanciano and Velasco, 2006). Therefore, we have estimated the *p*-value of the test statistics as the proportion of $D_n^{^\ast S2}$ which is higher than $D_n^2$. Moreover, we use a one-month window to estimate the evolving behavior of indices under study. Figures 4–6 exceptional exhibit levels of inefficiencies. The *p*-values reject the efficiency of returns and reveal that returns went under episodes of no predictability and significant predictability. Thus, we achieve the study’s first objective: returns from commodities exhibit evolving behavior. The solid line and dashed line on each graph in Figs. 4–6 present 5% and 10% significance levels for *p*-values, respectively. The evolving behavior of indices supports the notion of AMH of Lo (2004). It initiates the levels of predictabilities that we have further investigated by using linear and non-linear models under certain conditions of COVID-19.

### Linear tests

#### Autocorrelation

From linear tests, the most reliable and appropriate tool is the autocorrelation test to examine the independence of a series of returns (random variable). Usually, autocorrelation arises when different disturbances have non-zero correlations and covariances, i.e., for all $\ne j,$ ${\mathrm {Cov}}( {\varepsilon _i,\varepsilon _j} ) = \sigma _{ij}$, where $\varepsilon _t$ is the disturbance value in $i^{it}$ observation,

1 $$\rho _k = \frac{{\gamma _k}}{{\gamma _o}}$$

The positive-autocorrelation is inferred by $\rho \,>\, 0$, negative-autocorrelation is by $\rho \,<\, 0$, while no correlation is inferred by $\rho = 0,$ which indicates a random walk process and implies the null hypothesis of this test.

#### Runs test

Contrary to the autocorrelation test, the Runs test does not demand that a series should be normally distributed (Poshakwale, 1996). According to Siegel (1956), a run is a group of sequences or variables of similar value. We have computed the expected number of Runs as

2 $$E\left( \mu \right) = \frac{{2PN\left( {P + N} \right)}}{{\left( {P + N} \right)}} + 1$$

where *P* presents the positive number of runs and a negative number of runs by *N*. The variance of runs is computed by

3 $$\sigma ^2 = \frac{{2PN\left( {2PN - P - N} \right)}}{{\left( {P + N} \right)^2\left( {P + N - 1} \right)}}$$

The independence of a series of returns is the null hypothesis of this test. Once the *z*-value is greater than the critical values, we reject the null hypothesis.

#### Variance-ratio test

Lo and MacKinlay (1988) present the variance ratio (VR) test to gauge the predictability of asset prices to measure the variance of increments (RWI hypothesis) of a random walk (Hoque et al., 2007). The basic underlying assumption of this test is that the variance of *k* periods return is equivalent to the *k* times variance of a period in a random walk-in progression, showing the variance of returns from 10 days period is equivalent to 10 times-variance of its one-day return. Where $r_t\,{\mathrm {refers}}$ the VR test has *k* holding period. We have calculated it using the formula:

4 $${\mathrm {VR}}\left( k \right) = \frac{{\sigma _k^2}}{{k\sigma ^2}}$$

The $r_t$ infers the asset’s returns relevant to the *t* period, signifying *t* = 1, 2, 3….*T*. while for $k$ period, the variance is $\sigma _k^2$ = $r_t + r_{t - 1} + \ldots + r_{t - k + 1}$ is represented by

5 $${\mathrm {VR}}\left( k \right) = 1 + 2\mathop {\sum}\limits_{j = 1}^{k - 1} {\left( {1 - \frac{j}{k}} \right)\rho \left( j \right)}$$

where $\rho (j)$ signifies the $r_t$ Autocorrelation for *j* order and $1 + t$ is the variance ratio with increasing and decreasing weights of returns from assets. As $\rho \left( j \right) = 0$ showing zero correlation in a series of returns; hence, “the null hypothesis of variance ratio test is that: ${\mathrm {VR}}$ equals 1 for all $k{^{\prime}} s$”. Under the assumption of homoscedasticity, the null hypothesis $V\left( k \right) = 1,$ if $x_t$ is i.i.d. The test statistic $M_1\left( k \right)$ is given by

6 $$M_1\left( k \right) = \frac{{{\mathrm {VR}}\left( {x;k} \right) - 1}}{{\Phi (k)^{\frac{1}{2}}}}$$

The test statistic follows the standards asymptotically normal distribution, the asymptotic variance $\Phi \left( k \right)$ can be given by

7 $$\Phi \left( k \right) = \frac{{2(2k - 1)(k - 1)}}{{3k}}$$

As the returns exhibit conditional heteroskedasticity, Lo and MacKinlay (1988) accommodate this by proposing the robust heteroscedasticity test statistic $M_2\left( k \right);$

8 $$M_2\left( k \right) = \frac{{{\mathrm {VR}}\left( {x;k} \right) - 1}}{{\Phi \ast \left( k \right)^{1/2}}}$$

Under the null hypothesis $V\left( k \right) = 1,$ the test statistic asymptotically follows the standards of the normal distribution, where

9 $$\Phi \ast \left( k \right) = \mathop {\sum}\limits_{j = 1}^{k - 1} {\left[ {\frac{{2\left( {k - j} \right)}}{k}} \right]^2\delta \left( j \right)}$$

10 $$\delta \left(j \right) = \frac{{\big\{{\mathop{\sum}\nolimits_{t = j + 1}^T {({x_t - \hat \mu})^2\left({x_{t - j} - \hat \mu}\right)^2}} \big\}}}{{\left\{{\left[{\mathop{\sum}\nolimits_{t = 1}^T {\left({x_t - \hat \mu }\right)^2}}\right]^2}\right\}}}$$

We have applied the M2(*k*) test to a series of stock returns and standard normal distribution. We present results for 2, 4, 8, and 16 *k* holding periods.

### Non-linear tests

Earlier debate detects linear dependency in return series from commodities through conventional linear tests. Amini et al. (2010) report that in the absence of linear dependencies, the returns series still may have some non-linear serial dependencies that gained attention in the literature (Urquhart and Hudson, 2013; Ghazani and Araghi, 2014; Shahid et al., 2020a). Inherent nonlinearity is the primary characteristic of time series, so the following non-linear methods are more consistent to test the commodity markets’ efficiency by determining the levels of dependencies in the series compared to traditional linear methods (Alharbi, 2009). Therefore, we apply a pre-whitening AR model to remove the linearity from the series and apply a non-linear test on residuals to detect the non-linear dependency in the error term.

#### BDS Test

Brock et al. (1996) propose a portmanteau test: BDS, to spot time-varying dependencies in return for the series. This test uses the correlational dimensions of (Grassberger and Proceaccia, 1983) on a series with observation $\{ x_1 \ldots x_n\}$ and history of *m* such as $x_{mt} = (x_t,x_{t - 1}, \ldots \ldots \ldots x_{t - m + 1})$, while for $\varepsilon$ distance and “embedding dimension (*m*)*,”* we have computed the correlation integral {$C_m\left( {n,\varepsilon } \right)$} as

11 $$C_m\left( {n,\varepsilon } \right) = \frac{2}{{\left( {n - m} \right)\left( {n - m + 1} \right)}}\mathop {\sum}\limits_{S = 1}^{n - m} {\mathop {\sum}\limits_{t = S + 1}^{n - m + 1} {I_m\left( {x_S,x_t,\varepsilon } \right)} }$$

where sample size is represented by $n$ while any two observations possess the maximum difference $\varepsilon$ for any embedded dimension $m,$ which we have calculated during the computation of correlational integrals, the test statistic of the BDS is

12 $$W_m(\varepsilon ) = \sqrt {\frac{n}{{\hat V_m}}} \left( {C_m\left( {n,\varepsilon } \right) - C_1\left( {n,\varepsilon } \right)^m} \right)$$

where correlation integrals have a standard deviation of $\hat V_m$. With a normal distribution, $\sqrt {{{n}}} \left( {C_m\left( {n,\varepsilon } \right) - C_1\left( {n,\varepsilon } \right)^m} \right.$ is considered as a random variable in the BDS test; when $n$ increases, use ′*e*′ = $0.5\sigma ,1\sigma ,1.5\sigma \,{\mathrm {and}}\,2\sigma$ with a null hypothesis. According to Hsieh (1991), the leading cause of denial of ${\mathrm {H}}_0$ of BDS, i.e., ${\mathrm {i.i.d.}}$, is the presence of structural changes in the series of returns. We followed the metric bounds and embedding dimensions from 2 to 5 to a proportion of the standard deviation of the returns (Patterson and Ashley, 2000).

## Results

### Full sample period COVID-19

We have used linear tests to investigate the level of dependency/prediction, autocorrelation tests, runs test, and variance ratio tests. The autocorrelation test shows that from agriculture indices, only feeder cattle and live cattle indices show predictable returns based on historical trading. The autocorrelation statistic is significant at the 5% level (see Table 2). At the same time, similar behavior is shown by non-energy and crude oil from energy indices and silver, palladium, and platinum from precious metals at 5 Lags. The run test shows different results as the return from soybean from the agriculture sector is predictable. In contrast, none of the commodity returns from energy and precious metal indices are predictable based on past trading during the entire sample period of COVID-19.

**Table 2:** Predictability of returns for commodities is presented in columns 2–4 through linear tests, column 5 presents through BDS test (non-linear).

Full sample period COVID-19	Linear models			Non-linear model
	AC	RUNS test	VR	BDS test
	Lag 5		*K* = 4	5(2*σ*)
*Agriculture indices*				
Agr-Liv-Stock	−0.008*	−1.58	0.310050***	0.043712***
Agriculture	−0.014	−1.09	0.283657***	0.006829
Coffee	−0.046	0.03	0.253664***	0.030993***
Cotton	−0.001	0.89	0.251734***	0.042424***
Cocoa	−0.068	−0.24	0.247076***	4.63E−05
Corn	0.023	−1.56	0.294213***	0.0205488
Feeder cattle	−0.058***	−1.46	0.362167***	0.137354***
Live cattle	0.172**	−1.1	0.274747***	0.145516***
Soybean	0.023*	−2.07**	0.301340***	−0.001179
Sugar	0.059	−1.95*	0.243741***	0.031372***
Wheat	−0.006	1.71*	0.234760***	0.007737
*Energy indices*				
Red-Energy	0.016	−0.88	0.242919***	0.110082***
Light Energy	0.002	−1.07	0.254038***	0.100087***
Heating Oil	0.042	−0.69	0.171***	0.107022***
Petroleum	0.016	−0.44	0.216549***	0.141626***
Unld-Gaso	0.118	−0.76	0.228203***	0.082705***
Nonn-Gas	0.027	−0.88	0.235160***	0.114136***
Non-Ener	−0.058***	−0.88	0.293382***	0.059646***
Crude oil	−0.054***	−0.37	0.188257***	0.166066***
*Precious metals*				
Gold	−0.111	0.17	0.287262***	0.057936***
Metal	−0.123*	0.41	0.288343***	0.051860***
Silver	−0.123***	1.11	0.281064***	0.068655***
Copper	0.05	0.11	0.235355***	0.047866***
Palladium	−0.098***	−0.73	0.330764***	0.049625***
Platinum	−0.078**	1.93*	0.296606***	0.022757**
Ind-Metal	0.051	0.2	0.250171***	0.027779***
Nickel	−0.002	1.24	0.226257***	0.013901*
Zinc	−0.067	−0.95	0.281798***	−0.006615

AC & VR represent autocorrelation & variance ratio tests, respectively. *, ** & *** are the levels of significance at 10%, 5%, and 1%, respectively.

On the other hand, we obtain different results from the VR test at *K* = 4. Returns of almost all commodities from all three sectors under study are predictable during COVID-19 as the series exhibit linear dependencies in the returns (see test statistics are significant). We have applied the BDS test at the dimension of 5 and the embedded dimension of 2*σ* for the non-linear predictability. The majority of agriculture and precious metal indices are evidence of non-linear prediction because BDS statistics are significant at 1%. On the other hand, returns of all energy indices are strongly predictable during COVID-19.

### COVID-19 epidemic, pandemic, endemic & fatality phases, and AMH

The adaptive market hypothesis claims that returns from Financial markets go under periods of significant predictability and little predictability due to specific market conditions, bubbles, panics, and crises. Therefore, we divided the COVID-19 period into an epidemic, pandemic, endemic, and phases of fatality.

As for as different Covid phases are concerned, most indices go under periods of significant predictability and no predictability. Agriculture indices (except agriculture-livestock and live cattle) exhibit insignificant autocorrelation, which means there is no predictability of returns during the COVID-19 epidemic period, while returns are dependent and highly predictable during the pandemic (see Table 3). The behavior then reverts, and returns are not predictable in the endemic phase. Returns from all precious metals (except copper) and energy indices (except unleaded gasoline) show similar behavior. Returns from Live cattle are predictable in the pandemic and endemic phases but not in the epidemic phase. At the same time, both copper and unleaded gasoline are predictable in the epidemic and pandemic phases but not in the endemic phase, supporting AMH. With the Runs test, returns from soybean and sugar from agriculture, while most indices from the other two sectors go under periods of predictability and no predictability during COVID-19 market conditions. Thus, supporting AMH. At the variance ratio test, returns from the majority of indices are predictable in pandemic and endemic COVID-19 conditions while not predictable in the epidemic phase; hence, the results of linear tests support AMH.

**Table 3:** We present the adaptive behavior of commodity returns through COVID-19 epidemic and pandemic phases.

		Linear models			Non-linear model		Linear models			Non-linear model
		AC	Runs	VR	BDS		AC	Runs	VR	BDS
		Lag 5		*K* = 4	5(2*σ*)		Lag 5		*K* = 4	5(2σ)
Panel-A Agriculture sector indices						Panel-B Precious sector metals				
Agr-Liv-Stock	Epidemic	0.124	−0.68236	0.107517*	−0.001533	Gold	0.009	−0.3816	0.132826**	−0.016165
	Pandemic	−0.455	−0.72103	0.224782***	0.06945***		−0.475***	−0.5147	0.34532***	0.047267***
	Endemic	−0.016	−0.4	0.21563***	−0.009408		−0.169	1.32	0.272343***	0.013127
Agriculture	Epidemic	0.025	0.00752	0.189934*	0.039849***	Metal	0.015	−0.4321	0.173722**	−0.009516
	Pandemic	−0.399***	−1.3324*	0.269513***	0.037522***		−0.492***	−0.5064	0.249962	0.119538***
	Endemic	−0.12	−0.76	0.225375***	−0.00528		−0.154*	0.59	0.266296***	−0.002114
Coffee	Epidemic	−0.113	0.24809	0.152199*	−0.05605***	Silver	0.038	−1.0713	0.140201**	−0.016203
	Pandemic	−0.395***	−0.05022	0.304208	0.125295***		−0.564***	0.5168	0.230981***	0.017023
	Endemic	0.007	−0.79	0.269972***	−0.005686		−0.068*	1.96*	0.239183***	0.057368***
Cotton	Epidemic	0	−0.43216	0.136026**	0.031435	Copper	0.239**	−0.8236	0.11934**	0.014645
	Pandemic	−0.531***	0.73255	0.327796	0.244146***		−0.571***	−0.3439	0.305119***	0.043476***
	Endemic	0.034	1.24	0.265292***	−0.003434		0.003*	2.42**	0.213776***	0.004651
Cocoa	Epidemic	−0.003	−0.12974	0.123383	0.044402**	Palladium	0.027	−0.5696	0.224433**	0.020825
	Pandemic	−0.528***	−0.69684	0.299587***	0.112654***		−0.239**	−1.73**	0.253664***	0.065251***
	Endemic	−0.200*	−0.11	0.254279***	0.003865		−0.102	1.18	0.275815***	0.037723**
Corn	Epidemic	−0.14	0.70048	0.156963**	0.013537	Platinum	−0.207*	2.271*	0.127147*	0.026054*
	Pandemic	−0.295***	−1.08739	0.220689***	0.043805***		−0.555***	0.656	0.334241***	0.081232***
	Endemic	−0.144	−0.9	0.268656***	−0.01656		−0.177*	0.4	0.294932***	0.006572
Feeder Cattle	Epidemic	0.028	−0.24809	0.120834	−0.06057***	Ind-Metal	0.095	0.1924	0.125825*	0.05283***
	Pandemic	−0.278***	−0.28309	0.199351***	0.111291***		−0.572***	−0.5489	0.251232***	0.024258*
	Endemic	0.147	−0.9	0.2863***	−0.002138		0.008	1.2	0.246245***	−0.002114
Live Cattle	Epidemic	0.161	−0.49009	0.206533**	0.015434	Nickel	−0.074	0.0301	0.139057**	0.015632
	Pandemic	−0.424***	−1.3324*	0.281324	0.152473***		−0.56***	0.5720	0.286591	0.252822***
	Endemic	0.190**	−1.36	0.285844***	−0.027625		−0.01	0.4	0.212691***	−0.006154
Soybean	Epidemic	0.18	−2.144**	0.104027	−0.06158***	Zinc	−0.073	0.9059	0.12281	0.052059**
	Pandemic	−0.321***	−1.95***	0.339514	0.209613***		−0.399***	−0.7592	0.287154***	0.116662***
	Endemic	−0.067	0.23	0.241385***	−0.018776		−0.127*	−0.76	0.280422***	−0.013378**
Sugar	Epidemic	0.212*	−1.979**	0.155431**	−0.02503					
	Pandemic	−0.591***	−0.71298	0.299217***	0.103944***					
	Endemic	0.066	−0.5	0.192094***	−0.00197					
Wheat	Epidemic	0.045	0.03018	0.106328	−0.06158***					
	Pandemic	−0.525***	0.3195	0.190192***	0.156291***					
	Endemic	−0.125	0.98	0.198059***	0.014101					
Panel-C Energy sector indices										
Red-Energy	Epidemic	0.179	−2.9183***	0.112586**	−0.015985	Unld-Gaso	0.336***	−2.217**	0.179371**	0.006132
	Pandemic	−0.574***	−1.01703	0.283022***	0.042449**		−0.564***	0.30098	0.291208	0.072756***
	Endemic	0.023	−0.07	0.281893***	0.0136**		0.045	0.51	0.304126***	0.013736**
Light energy	Epidemic	0.209*	−3.0572***	0.134046	0.047893**	Nonn-Gas	0.157	−2.293**	0.13307	−0.05853***
	Pandemic	−0.607***	−0.49008	0.205855***	0.117572***		−0.536***	−0.25765	0.314704	0.109508***
	Endemic	−0.017	0.2	0.262585***	−0.002092		0.064	−0.07	0.289254***	0.022158
Heating oil	Epidemic	0.06	−1.51697*	0.13312	0.024777	Non-Ener	0.214*	−0.88531	0.12953**	−0.00036
	Pandemic	−0.475***	0.33753	0.34065***	0.093363***		−0.579***	−0.7739	0.249757***	0.005228
	Endemic	0.059	−0.73	0.265282***	0.024970*		−0.102	0.29	0.220991***	−0.017198
Petroleum	Epidemic	0.102	−2.47374**	0.157787**	−0.033314**	Crude oil	0.044	−2.99***	0.122827**	−0.010886
	Pandemic	−0.481***	0.77951	0.45092***	0.04311		−0.486***	0.57208	0.338264***	0.036779***
	Endemic	0.089	0.4	0.292890***	0.056968		0.095	−0.19	0.286468***	0.064357***

The Epidemic phase ranges from the emergence of the Coronavirus to March 10, 2020. The Pandemic Period ranges from March 10, 2020, to date as per WHO. The varying degree of the behavior of returns from Commodities is presented in columns 3–5 through linear tests, while column 6 presents the varying levels of predictability through the BDS test. Column 2 Presents sub-samples phases (Epidemic & Pandemic). *, ** & *** presents levels of significance at 1%, 5%, & 10%, respectively. We represent autocorrelation by AC and variance ratio by VR.

Results of the non-linear test reveal that commodities^6^ exhibit significant non-linear prediction (at 5%) during the pandemic while no non-linear prediction during both epidemic and endemic phases with the application of the BDS test (see Table 3). Returns from commodities^7^ exhibit significant non-linear prediction during epidemic and pandemic while no non-linear prediction during the endemic phase. In contrast, returns from palladium, reduced energy, and unleaded gasoline are not predictable in epidemic conditions while predictable in pandemic and endemic conditions of COVID-19. The result from non-linear BDS tests strongly support AMH and are similar to the studies (Urquhart and Hudson, 2013: Ghazani and Araghi, 2014; Shahid et al., 2019a).

We observe pretty exciting results from the fatality phases of Covid-19 because the majority of the commodities go under periods of predictability and no predictability, thus, supporting AMH. The results of the autocorrelation test reveal that returns of agriculture livestock, agriculture index, feeder cattle, live cattle, and wheat are not predictable. Also, heat oil, non-energy oil, gold, metal, silver, palladium, nickel, and zinc from metal indices are not predictable in the first two consecutive phases (fatal1 & fatal2) while predictable in the third phase of fatalities (fatal3). Our results show similar behavior for soybean, palladium, & platinum at runs test, silver at VR test, and cotton, corn, sugar, unleaded gasoline, & nickel with non-linear BDS test. However, unleaded gasoline with the VR test and coffee, cocoa, live cattle, soybean, wheat, petroleum, & oil with the BDS test remain unpredictable in the fatal1 period while predictable in the 2 & 3 phases. The commodity indices (corn, soybean, reduced energy, petroleum, unleaded gasoline, non-natural gasoline, platinum) show significant predictability in phase fatal1 and unpredictable in phase fatal2, then again predictable in phase fatal3. Commodities show similar behavior^8^ on the V.R. test. While the rest of the commodity indices also go under periods of significant predictability in certain Fatal phases and unpredictability in other Fatal phases, thus supporting AMH. We support our results at 1% and 5% confidence levels (see Table 4). Finally, we show that certain market conditions produce profitable opportunities as produced by epidemic, pandemic, and fatality phases. Therefore, we achieve the second objective, the study that evolving behavior of markets produces a prediction for profitable opportunities during COVID-19. Moreover, our results are similar to the studies of (Ramírez et al., 2015; Shahid et al., 2019c) that markets evolve under certain conditions or crises.

**Table 4:** We present the adaptive behavior of commodity returns through COVID-19 fatality phases.

Panel-1						Panel-2				
Sub-sample phases	Energy sector indices	Linear models			Non-linear model	Agriculture sector indices	Linear models			Non-linear model
		Auto	Runs	VR	BDS		Auto	Runs	VR	BDS
		Lag 5		*K* = 4	5(2*σ*)		Lag 5		*K* = 4	5(2*σ*)
Fatal1	Red-Energy	0.355***	−0.561	0.688**	0.0376**	Agr-Liv-Stock	0.073	0.0114	0.426***	0.110***
Fatal2		−0.481*	−2.128**	0.570	0.0255*		−0.489*	0.0401	0.540	−0.10***
Fatal3		−0.539***	−0.413	0.444***	0.0212		−0.432***	−0.792	0.635***	0.017***
Fatal1	Light Energy	0.377***	−0.529	0.409***	0.0405**	Agriculture	−0.071	0.3336	0.468***	0.028*
Fatal2		−0.561**	−2.128**	0.820	−0.062***		−0.454	1.2247	0.573	−0.014
Fatal3		−0.563***	−0.906	0.584**	0.0181***		−0.377***	−1.389*	0.578***	0.014
Fatal1	Heating Oil	0.127	−0.561	0.639**	−0.017	Coffee	−0.193	−0.274	0.413***	−0.049*
Fatal2		−0.443	−1.224	0.738	0.0752***		−0.802***	1.4859*	0.210**	0.1109***
Fatal3		−0.468***	0.2349	0.512***	0.0196*		−0.32***	−0.137	0.481***	0.0191***
Fatal1	Petroleum	0.274**	−1.866**	0.428***	0.0078	Cotton	−0.253**	0.9745	0.239***	0.0277
Fatal2		−0.153	−2.128**	0.737	−0.198***		−0.573**	−2.12**	0.239*	0.0089
Fatal3		−0.475***	0.70141	0.443***	0.0136***		−0.516***	0.81294	0.691***	0.0165***
Fatal1	Unld-Gaso	0.303**	−1.714**	0.698*	−0.0038	Cocoa	−0.063	−0.5292	0.471***	−0.0037
Fatal2		0.159	−2.128**	0.737	−0.0267		−0.666**	0.76305	0.301*	−0.219***
Fatal3		−0.543***	1.1219	0.496***	0.0249***		−0.527***	−0.7924	0.432***	0.0199***
Fatal1	Nonn-Gas	0.327**	−1.133	0.445***	0.0075	Corn	−0.287**	1.06813	0.431**	0.0303*
Fatal2		−0.342	−2.128**	0.519	0.0867***		−0.295	0	0.494**	−0.0266
Fatal3		−0.512***	−0.4137	0.470***	0.0157		−0.274***	−1.194	0.740**	0.0140**
Fatal1	Non-Ener	0.263*	−1.3922*	0.618**	−0.0228	Feeder cattle	0.041	−0.8169	0.335**	0.0606**
Fatal2		−0.569*	0.04016	0.951	0.1938***		−0.372	−0.6827	0.268*	0.0867***
Fatal3		−0.536***	−0.8363	0.522**	0.0141		−0.311***	0.03524	0.477***	0.0210***
Fatal1	Crude Oil	0.244*	−1.1917	0.480***	−0.026	Live cattle	−0.081	0.62133	0.358***	0.0069
Fatal2		−0.041	−2.128**	0.723*	0.1466***		−0.361	0.04016	0.820	0.0969***
Fatal3		−0.488***	0.142474	0.777*	0.0151***		−0.423***	−0.9915	0.697**	0.0181***
Precious Metal Indices	Soybean	0.276**	−1.392*	0.623**	0.0387					
Fatal1	Gold	0.095	0.333	0.665523**	0.015636		−0.547*	−0.6827	0.751	0.1109***
Fatal2		−0.188	−0.408	0.842488	0.110969***		−0.349***	−1.9***	0.527***	0.0262***
Fatal3		−0.48***	−0.195	0.534805***	0.015831*	Sugar	0.151	−0.561	0.624**	0.0198
Fatal1	Metal	0.091	0.3336	0.258307***	0.024407		−0.71***	0.04016	0.599	−0.0266*
Fatal2		−0.204	−0.408	0.793115	0.039541*		−0.584***	−0.9915	0.541***	0.0225***
Fatal3		−0.496***	−0.597	0.446822***	0.016197*	Wheat	0.097	−0.2857	0.459***	0.0099
Fatal1	Silver	−0.052	0.5958	0.714731*	−0.035316**		−0.216	−0.4082	0.330*	−0.154***
Fatal2		−0.303	−2.128**	0.643877	−0.11734***		−0.525***	0.8002	0.435***	0.0227***
Fatal3		−0.562***	0	0.468404***	0.014497					
Fatal1	Copper	0.311**	−2.453***	0.599395**	0.065982***					
Fatal2		−0.698**	0.763	0.663618	0.003827					
Fatal3		−0.566***	−0.084	0.446915***	0.014152***					
Fatal1	Palladium	−0.059	−0.105	0.566589**	0.065179***					
Fatal2		−0.254	0.4082	0.568042	−0.010204					
Fatal3		−0.237**	−1.977***	0.661146***	0.017671**					
Fatal1	Platinum	−0.371***	2.9225***	0.775455***	0.05294**					
Fatal2		−0.332	0.0401	0.293208*	0.086416**					
Fatal3		−0.561***	1.1516	0.421128***	0.015114***					
Fatal1	Ind-Metal	0.129	−0.595	0.565581***	0.089756***					
Fatal2		−0.659**	0.763	1.383749	0.003827					
Fatal3		−0.557***	−0.261	0.465087***	0.014402*					
Fatal1	Nickel	−0.073	−0.185	0.536172***	−0.038834*					
Fatal2		−0.333	0	0.714467*	0.003827					
Fatal3		−0.536***	0.8	0.475156***	0.018869***					
Fatal1	Zinc	−0.057	0.333	0.466287***	0.045131**					
Fatal2		−0.5*	0.763	0.564231	−0.21938***					
Fatal3		−0.404***	−0.906	0.591722***	0.020071***					

We divide the fatalities into three phases, Fatal1, which ranges from January 1, 2020, to February 14, 2020 (when deaths mainly occur in China only). The second phase, Fatal2, ranges from February 15 to 28, 2020 (when fatalities start occurring in Europe), and Fatat3 ranges after post Fatal2 to date when deaths occur in the USA and around the globe. Column 1 Presents sub-samples phases (Fatal1, Fatal2 & Fatal3). *, ** & *** presents levels of significance at 1%, 5%, & 10%, respectively.

## Conclusion

This study explores the varying degree of commodity markets through AMH because AMH absorbed or engaged in the changing behavior of returns during the epidemic, pandemic, endemic, and fatality shocks (phases) of COVID-19. We show that the AMH explains the response of commodity markets to COVID-19 sub-phases. Using daily data of well-known and most commonly traded commodities from the Chicago Board of Options Exchange (CBOE) of the U.S., we apply the Generalized Spectral (GS) test to detect the adaptive behavior of commodity markets during COVID-19. We also apply linear and non-linear econometric models to identify whether evolving behavior of markets produces a prediction for profitable opportunities during COVID-19.

We find positive returns in most commodities understudy during the COVID-19 period. In a more detailed analysis, during the full-sample COVID-19 period, few commodities are predictable with autocorrelation and the runs test, while most commodities are more predictable with linear VR and non-linear BDS test, indicating signs of the inefficiency of commodity markets. Most indices from all three sectors are not predictable in epidemic and endemic periods, while this behavior is reversed in the pandemic. Similarly, indices are predictable in certain COVID-19 conditions (epidemic, pandemic, and endemic) while not predictable in others with linear and non-linear econometric models. On the other hand, returns from commodities are significant during some of the COVID-19 fatality phases while insignificant in others. Overall, our analysis reveals that commodity markets swiftly respond to the fatal3 and pandemic phases of COVID-19.

In summary, returns from commodities exhibit time-varying behavior during COVID-19, and this changing behavior produces profitable opportunities under certain COVID-19 conditions in the market. Thus, the behavior of COVID-19 shocks is engaged and best explained by the adaptive market hypothesis (AMH), which claims that returns from financial markets go under periods of significant predictability and little predictability due to specific market conditions, bubbles, panics, and crises. Finally, we enhance the literature on COVID-19 by first time exploring the behavior engagement of commodities’ return with COVID-19 under the umbrella of AMH. We propose undertaking this study for debt, equity, or currency markets and leave it to aspiring researchers.

## Data Availability

The datasets generated and analyzed during the current study are available in the Investing database [https://www.investing.com/].

## Footnotes

1 Fama’s (1970) notion of market efficiency suggests a lack of predictability of return.

2 ADX of Abu index of Dhabi, CAC40 index of France, GDAXI index of Germany, DJIA index of The USA, FTSE100 index of UK, KLSE index of Malaysia, JKSE index of Indonesia, KOSPI index of Korea, IMOEX.ME index of Russia, N225 index of Japan, AXJO index of Australia, GSPTSE index of Canada, STI index of Singapore, TPE TAIEX index of Taiwan, AAXJ index of Asia ex Japan, SET50 index of Thailand, HSI index of Hong Kong, SSEC index of Shanghai, SZCS index of Shenzhen, FTMIB index of Italy, NIFTY-50 index of India.

3 UK, Spain, France, Italy, Iran, China.

4 From Asia: Japan, Korea, China, India.

5 From Europe: Italy, France, Germany, Netherlands, Switzerland.

6 Agriculture live stock, cotton, corn, live cattle, sugar, gold, metal, copper, platinum, nickle & heating oil.

7 Agriculture, coffee, cocoa, feeder cattle, soybean, wheat, light energy, and non-natural gas.

8 Agriculture Livestock, Agriculture Index, Cotton, Cocoa, Feeder Cattle, Live Cattle, Soybean, Sugar, Wheat, Gold, Metal, Non-energy, Non-natural Gas, Reduced Energy, Petroleum, Light energy, Cooper, Palladium, Platinum, Industrial Metal, Nickel, Zinc.

## References

[CR1] Al-Awadhi AM, Alsaifi K, Al-Awadhi A, Alhammadi S (2020). Death and contagious infectious diseases: Impact of the COVID-19 virus on stock market returns.. J Behav Exp Finance.

[CR2] Alharbi AM (2009) Nonlinearity and market efficiency in GCC stock markets. Doctoral dissertation, University of Kansas

[CR3] Al Refai H, Zeitun R, Eissa MAA (2022). Impact of global health crisis and oil price shocks on stock markets in the GCC.. Finance Res Lett.

[CR4] Ali M, Alam N, Rizvi SAR (2020). Coronavirus (COVID-19)—An epidemic or pandemic for financial markets. J Behav Exp Finance.

[CR5] Alfaro L, Chari A, Greenland AN, Schott PK (2020) Aggregate and firm-level stock returns during pandemics, in real-time (No. w26950). National Bureau of Economic Research

[CR6] Amini S, Hudson R, Keasey K (2010). Stock return predictability despite low autocorrelation. Econ Lett.

[CR7] Ashraf BN (2020). Stock markets’ reaction to COVID-19: Cases or fatalities?. Res Int Bus Finance.

[CR8] Bai Y (2014). Cross-border sentiment: an empirical analysis on E.U. stock markets. Appl Financ Econ.

[CR9] Baker M, Wurgler J, Yuan Y (2012). Global, local, and contagious investor sentiment. J Financ Econ.

[CR10] Baker SR, Bloom N, Davis SJ, Terry SJ (2020) Covid-induced economic uncertainty (No. w26983). National Bureau of Economic Research

[CR11] Brock WA, Scheinkman JA, LeBaron B, Dechert WD (1996). A test for independence based on the correlation dimention. Econom Rev.

[CR12] Chen MH, Jang SS, Kim WG (2007). The impact of the SARS outbreak on Taiwanese hotel stock performance: an event-study approach. Int J Hosp Manag.

[CR13] Chen MP, Lee CC, Lin YH, Chen WY (2018). Did the SARS epidemic weaken the integration of Asian stock markets? Evidence from smooth time-varying cointegration analysis. Econ Res-Ekon Istraž.

[CR14] Del-Giudice A, Paltrinieri A (2017). The impact of the Arab Spring and the Ebola outbreak on African equity mutual fund investment decisions. Res Int Bus Finance.

[CR15] Dunford D, Dale B, Stylianou N, Lowther E, Ahmed M, De la Torres Arenas I (2020) Coronavirus: the world in lockdown in maps and charts. BBC News [S.l.]

[CR16] Escanciano JC, Velasco C (2006). Generalized spectral tests for the martingale difference hypothesis. J Econom.

[CR17] Fama FE (1970). Efficient capital markets: a review of theory and empirical work. J Finance.

[CR18] Ghazani MM, Ebrahimi SB (2019). Testing the adaptive market hypothesis as an evolutionary perspective on market efficiency: evidence from the crude oil prices. Finance Res Lett.

[CR19] Grassberger P, Proceaccia I (1983). Measuring the strangeness of strange attractors. Physica: Non-linear Phenom.

[CR20] Gates B (2020). Responding to Covid-19—a once-in-a-century pandemic?. N Engl J Med.

[CR21] Ghazani MM, Araghi MK (2014). Evaluation of the adaptive market hypothesis as an evolutionary perspective on market efficiency: evidence from the Tehran stock exchange. Res Int Bus Finance.

[CR22] Gunay S (2021). Comparing COVID-19 with the GFC: a shockwave analysis of currency markets. Res Int Bus Finance.

[CR23] Goodell JW (2020) COVID-19 and finance: agendas for future research. Finance Res Lett. 101512. 10.1016/j.frl.2020.101512 PMC7152896 32562472

[CR24] Haroon O, Rizvi SAR (2020). COVID-19: Media coverage and financial markets behavior—A sectoral inquiry.. J Behav Exp Finance.

[CR25] Hoque HA, Kim JH, Pyun CS (2007). A comparison of variance ratio tests of random walk: a case of Asian emerging stock markets. Int Rev Econ Finance.

[CR26] Hsieh DA (1991). Chaos and nonlinear dynamics: application to financial markets. J Finance.

[CR27] Lee JW, McKibbin WJ (2004). Globalization and disease: the case of SARS. Asian Econ Pap.

[CR28] Li Z, Ge J, Yang M, Feng J, Qiao M, Jiang R, Yang C (2020). Vicarious traumatization in the general public, members, and non-members of medical teams aiding in COVID-19 control.. Brain Behav Immun.

[CR29] Lin B, Su T (2021). Does COVID-19 open Pandora’s box of changing the connectedness in energy commodities?. Res Int Bus Finance.

[CR30] Liu H, Manzoor A, Wang C, Zhang L, Manzoor Z (2020). The COVID-19 outbreak affected countries’ stock markets response. Int J Environ Res Public Health.

[CR31] Lo AW (2004). The adaptive market hypothesis. J Portf Manag.

[CR32] Lo AW, MacKinlay AC (1988). Stock market prices do not follow random walks: evidence from a simple specification test. Rev Financ Stud.

[CR33] Macciocchi D, Lanini S, Vairo F, Zumla A, Moraes Figueiredo LT, Lauria FN, Kremsner P (2016). The short-term economic impact of the Zika virus outbreak. New Microbiol.

[CR34] Matos P, Costa A, da Silva C (2021). COVID-19, stock market and sectoral contagion in the U.S.: a time–frequency analysis. Res Int Bus Finance.

[CR35] Papadamou S, Fassas A, Kenourgios D, Dimitriou D (2020) Direct and indirect effects of COVID-19 pandemic on implied stock market volatility: evidence from panel data analysis. MPRA Paper 100020, University Library of Munich, Germany.

[CR36] Patterson D, Ashley RA (2000) A nonlinear time series workshop: a toolkit for detecting and identifying nonlinear serial dependence. Kluwer Academic, Boston, MA.

[CR37] Okorie DI, Lin B (2021). Stock markets and the COVID-19 fractal contagion effects.. Finance Res Lett.

[CR38] Okorie DI, Lin B (2021). Adaptive market hypothesis: the story of the stock markets and COVID-19 pandemic. N Am J Econ Finance.

[CR39] Onali E (2020) Covid-19 and stock market volatility. Available at SSRN 3571453.

[CR40] Poshakwale S (1996). Evidence on weak form efficiency and day of the week effect in the Indian Stock Market. Finance India.

[CR41] Ramírez SC, Arellano PLC, Rojas O (2015). Adaptive market efficiency of agricultural commodity futures contracts. Contad Adm.

[CR42] Shahid MN, Mehmood Z (2015). Calendar anomalies in stock market: a case of KSE 100 index. Int J Afr Asian Stud.

[CR43] Shahid MN, Sattar A (2017). Behavior of calendar anomalies, market conditions and adaptive market hypothesis: evidence from Pakistan stock exchange. Pak J Commer Soc Sci.

[CR44] Shahid MN, Jabeen S, Sattar A, Ateeq A (2018). Behavior of bitcoin returns and adaptive market hypothesis (AMH). Asia Pac J Emerg Mark.

[CR45] Shahid MN, Coronado S, Sattar A (2019). Stock market behaviour: efficient or adaptive? Evidence from the Pakistan Stock Exchange. Afro-Asian J Finance Account.

[CR46] Shahid MN, Jehanzeb M, Abbas A, Zubair AAkbar MAH (2020) Predictability of precious metals and adaptive market hypothesis Int J Emerg Mark 15

[CR47] Shahid MN, Latif K, Chaudhary GM, Adil S (2020). Financial crises and adaptive market hypothesis: an evidence from International Commodities traded at New York Stock Exchange. Rev Econ Dev Stud.

[CR48] Shahid MN, Latif K, Chaudhary GM, Kouser R (2020). Vacillating behavior of TOM effect and adaptive market hypothesis: a firm level evidence from emerging stock market of Pakistan. J Bus Soc Rev Emerg Econ.

[CR49] Siegel S (1956). Non-parametric statistics for the behavioural sciences.

[CR51] Urquhart A, Hudson R (2013). Efficient or adaptive markets? Evidence from major stock markets using very long-run historic data. Int Rev Financ Anal.

[CR52] Wagner AF (2020). What the stock market tells us about the post-COVID-19 world. Nat Hum Behav.

[CR53] Zaremba A, Aharon DY, Demir E, Kizys R, Zawadka D (2021). COVID-19, government policy responses, and stock market liquidity around the world: a note. Res Int Bus Finance.

[CR54] Zhang D, Hu M, Ji Q (2020). Financial markets under the global pandemic of COVID-19.. Finance Res Lett.

